# SPP1 Promotes Enzalutamide Resistance and Epithelial-Mesenchymal-Transition Activation in Castration-Resistant Prostate Cancer via PI3K/AKT and ERK1/2 Pathways

**DOI:** 10.1155/2021/5806602

**Published:** 2021-10-22

**Authors:** Xiaocong Pang, Junling Zhang, Xu He, Yanlun Gu, Bin-zhi Qian, Ran Xie, Wei Yu, Xiaodan Zhang, Teng Li, Xuedong Shi, Ying Zhou, Yimin Cui

**Affiliations:** ^1^Department of Pharmacy, Peking University First Hospital, Beijing 100034, China; ^2^Department of General Surgery, Peking University First Hospital, Beijing 100034, China; ^3^MRC Centre for Reproductive Health, College of Medicine and Veterinary Medicine, Queen's Medical Research Institute, The University of Edinburgh, Edinburgh, UK; ^4^Department of Urology, Peking University First Hospital, Beijing 100034, China; ^5^Department of Medical Oncology, National Clinical Research Center for Cancer, Chinese Academy of Medical Sciences and Peking Union Medical College, Beijing 100021, China; ^6^Department of Orthopedics, Peking University First Hospital, Beijing 100034, China

## Abstract

The bottleneck arising from castration-resistant prostate cancer (CRPC) treatment is its high metastasis potential and antiandrogen drug resistance, which severely affects survival time of prostate cancer (PCa) patients. Secreted phosphoprotein 1 (SPP1) is a cardinal mediator of tumor-associated inflammation and facilitates metastasis. In our previous study, we firstly revealed SPP1 was a potential hub signature for predicting metastatic CRPC (mCRPC) development. Herein, we integrated multiple databases to explore the association of SPP1 expression with prognosis, survival, and metastatic levels in CRPC progression and investigated SPP1 expression in PCa tissues and cell lines. Next, PCa cell lines with overexpression or depletion of SPP1 were established to study the effect of SPP1 on enzalutamide sensitivity and adhesion and migration of prostate cancer cell lines and further explore the underlying regulatory mechanisms. Bioinformatics analysis, polymerase chain reaction (PCR), immunohistochemical staining, and western blot results suggested SPP1 upregulation had strong relationship with the malignant progression of CRPC and enzalutamide resistance. SPP1 knockdown enhanced enzalutamide sensitivity and repressed invasion and migration of prostate cancer cells. Importantly, upregulating SPP1 promoted, while silencing SPP1 attenuated epithelial-mesenchymal-transition (EMT). Our results further demonstrated that SPP1 overexpression maintains the activation of PI3K/AKT and ERK1/2 signaling pathways. Overall, our findings unraveled the functional role and clinical significance of SPP1 in PCa progression and help to discover new potential targets against mCRPC.

## 1. Introduction

Prostate cancer (PCa) is the most common malignancy, which causes the fifth rank of cancer-related mortality and is approximated 1,410,000 deaths and accounts for almost 14% of total cancer diagnosed in men [1, 2]. A significant feature of PCa is its hormone responsiveness, initially identified by Huggins and Hodges in 1941, who recognized that castration caused tumor regression in PCa patients [3]. Androgen deprivation therapy (ADT) using androgen receptor (AR) blockers to inhibit the androgen pathway is current standard therapy for PCa [3]. In spite of the high long-term survival in localized PCa, antiandrogen treatment resistance can lead to primary castration-resistant prostate cancer (CRPC) or even metastatic CRPC (mCRPC) [4]. PCa is still a critical medical problem for the males affected, with excessive medical treatment of inherently benign disease and deficient effective treatment for metastatic PCa [5]. The underlying mechanisms of metastasis and antiandrogen treatment resistance of PCa are still not completely cleared.

Secreted phosphoprotein 1 (SPP1), also recognized as osteopontin (OPN), a secreted chemokine-like glycophosphoprotein, is a cardinal mediator of tumor-associated inflammation and facilitates metastasis [6]. SPP1 is a crucial extracellular matrix component, secreted by multiple kinds of cell types including tumor cells, immune cells, fibroblasts, osteoclasts, smooth muscle, lymphocytes, and epithelial cells [7]. Upregulation of SPP1 in tumor tissue and plasma was found to be associated with poor prognosis in patients in many kinds of cancer [8–10]. In PCa cells, it was reported that SPP1 could upregulate p-glycoprotein expression to induce multidrug resistance [11]. In a prospective cohort of breast cancer patients received adjuvant chemotherapy, the SPP1 expression level could predict the efficiency of neoadjuvant chemotherapy in certain patients [12]. In our previous study, we firstly revealed that SPP1 was a potential hub signature for predicting mCRPC development [13], but the association between SPP1 overexpression and antiandrogen treatment resistance or metastasis needs to be further investigated.

Under certain physiological and pathological conditions, epithelial cells differentiate into mesenchyme, accompanied by changes in cell morphology and related genes [14], which is called epithelial-mesenchymal-transition (EMT). Emerging evidence demonstrates that the EMT process can promote tumor cell invasion and metastasis by loss of cell-cell connections and cell polarity [15]. The characteristic manifestations of EMT are mainly epithelial cell phenotype-specific proteins such as E-cadherin and claudin-1 decreased, and mesenchymal cell phenotype-specific proteins such as N-cadherin and vimentin increased. There are also explicit evidences indicating that EMT may be an important driving factor leading to drug resistance and disease recurrence [15]. With antiandrogen agent enzalutamide treatment, PCa cells undergo EMT changes, referring to the reduction of the expression of epithelial markers and overexpression of mesenchymal markers [16]. Despite of some findings about EMT in CRPC, the regulatory mechanism via extracellular stimulus by which regulates EMT in CRPC progression remains to be elucidated [17, 18].

In this study, we firstly integrated multiple databases, including TCGA, GEO, UALCAN, and HCMDB, to demonstrate the relationship of SPP1 expression with PCa prognosis, survival, metastatic levels, and CRPC progression. Next, we examined the effect of the regulation of SPP1 on enzalutamide sensitivity, migration, and adhesion of PCa cell lines, which had been genetically modified to overexpress or deplete SPP1. Moreover, we further explored the functional role of SPP1 in EMT activation and the underlying regulatory mechanisms. Overall, this study is aimed at identifying the clinical significance and biological functions of SPP1 in CRPC progression and helps in identifying novel potential biomarker and therapeutic strategy.

## 2. Results

### 2.1. SPP1 As an Important Signature in Malignant CRPC Progression

**Figure 1:**
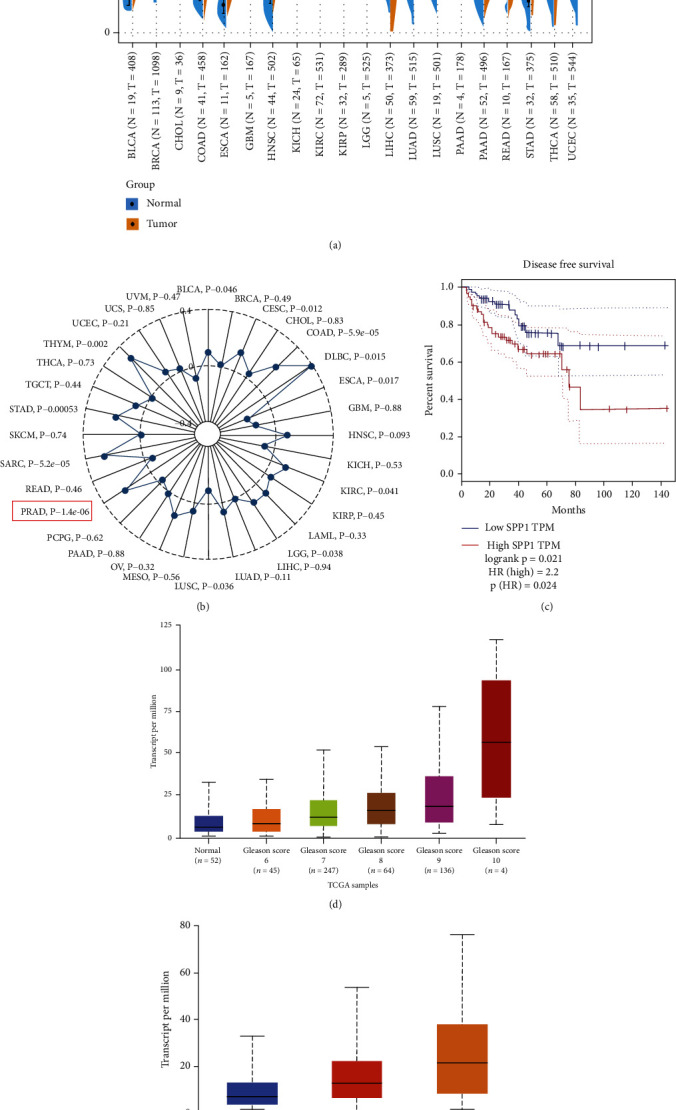
High expression of SPP1 in prostate cancer and its relationship with survival, Gleason score, and metastatic levels. (a) SPP1 expression was analyzed in pan-cancer and was found overexpressed in multiple cancers, including PCa, breast cancer, colorectal cancer, and lung adenocarcinoma. (b) Tumor mutational burden (TMB) analysis in pan-cancer. The expression level of SPP1 was mostly correlated with TMB level with *P* value as 1.4*e* − 06 in PCa. (c) Disease-free survival (DFS) was significantly lower in patients who have high expression of SPP1. (d) SPP1 expression in PCa was positively associated with clinical stages. (e) SPP1 expression in PCa had a good relationship with lymph node metastasis. N0: no regional lymph node metastasis; N1: metastases in 1 to 3 axillary lymph nodes.

**Figure 2:**
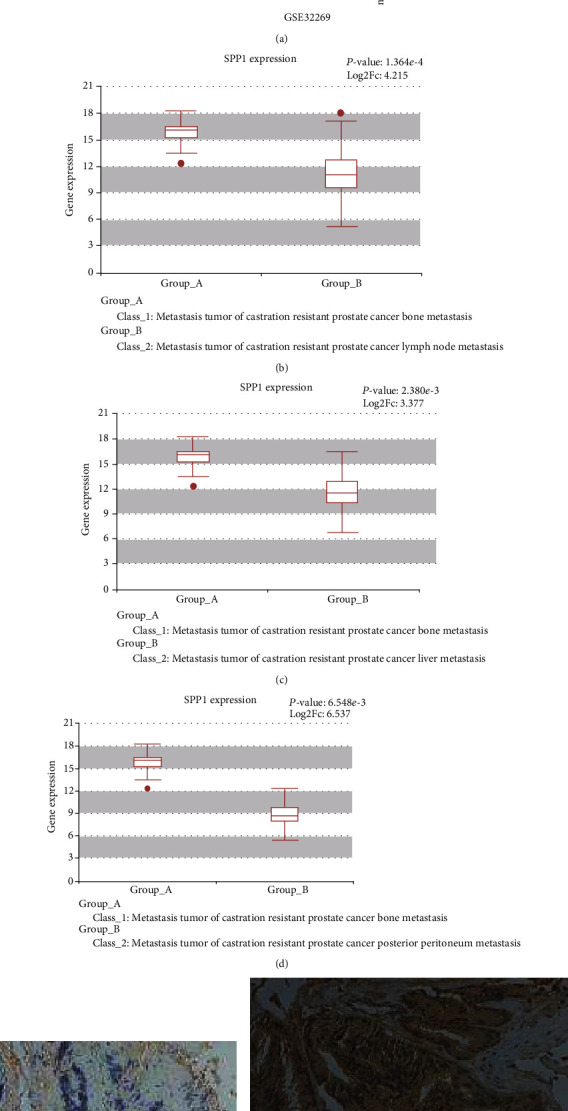
High expression of SPP1 in CRPC with bone metastasis. (a) Comparison of SPP1 expression in mCRPC group and PCa group in GSE32269 dataset. (b–d) HCMDB analysis of SPP1 expression level in bone metastasis compared in lymph nodes and posterior peritoneum metastasis. Immunohistochemical (IHC) staining performed to determine the expression of SPP1 in primary PCa (e) and bone metastasis CRPC (f).

SPP1 as the important extracellular matrix component was found overexpression in many kinds of tumors, including PCa, breast cancer, colorectal cancer, and lung adenocarcinoma (Figure 1(a)). Tumor mutational burden (TMB) is generally defined as the total number of somatic mutations in per million bases (Mb). Recently, it was reported that TMB in the lethal mCRPC clinical state, which was not controlled by androgen ablation, was significantly increased, and also had a good relationship with PCa patients' advancing clinical state and Gleason score [18]. In PCa, we found the expression level of SPP1 mostly correlated with TMB level with *P* value as 1.4*e* − 06 (Figure 1(b)). In addition, disease-free survival (DFS) was significantly lower in patients who were highly expressed SPP1 (*P* value = 0.021, HR = 2.2) (Figure 1(c)), whereas the metastasis-free survival was not significantly associated with SPP1 expression (*P* = 0.305) (Supplement Figure 1). The expression level of SPP1 in PCa was positively associated with clinical stages and lymph node metastasis (Figures 1(d) and 1(e)). In GSE32269 dataset, SPP1 expression in the mCRPC group was significantly higher than that in the PCa group (Figure 2(a)). HCMDB analysis showed that SPP1 expression level in bone metastasis was remarkably higher than that in lymph node and posterior peritoneum metastasis (Figures 2(b)–2(d)), which suggested SPP1 was associated with distant metastases. Conformably, immunohistochemistry (IHC) staining results also demonstrated SPP1 was higher expressed in CRPC with bone metastasis group compared with primary PCa tissues (Figures 2(e) and 2(f)). Therefore, the expression of SPP1 has a strong relationship with the progression of CRPC.

### 2.2. Expression of SPP1 in Different Derived PCa Cells and CRPC Tissues

**Figure 3:**
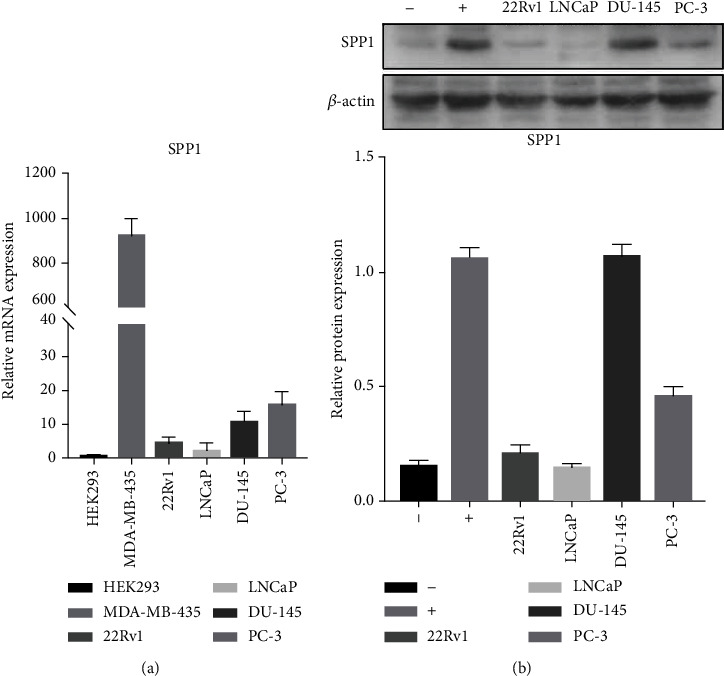
Expression of SPP1 in different derived PCa cells. (a). RNA expression level in four groups of cell lines (PC-3, DU-145, LNCaP, and 22Rv1) with SPP1 negative and positive expression cells as control groups. (b). Protein expression level in PC-3, DU-145, LNCaP, and 22Rv1 with SPP1 negative (-) and positive (+) expression cells as control groups.

To reveal the differential expression of SPP1 in PCa cells, we detected the RNA and protein expression level in four groups of cell lines (PC-3, DU-145, LNCaP, and 22Rv1) with SPP1 negative and positive expression cells as control groups. As shown in Figure 3, these results demonstrated that 22Rv1 as AR-positive and enzalutamide-resistant PCa cell line has higher RNA and protein expression of SPP1 than that in LNCaP cell line which is AR-positive and androgen-dependent. In addition, the RNA and protein level of SPP1 were highest in both DU-145 and PC-3 cell lines, which were AR-negative and androgen-independent metastatic CRPC cell lines. Therefore, SPP1 was higher expressed in androgen-independent and metastatic PCa cell lines.

**Figure 4:**
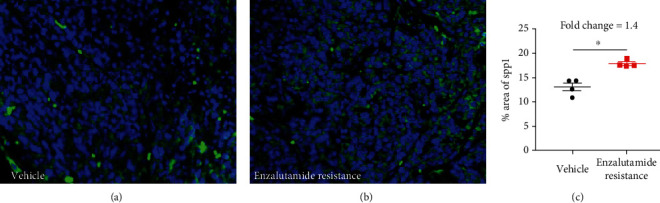
Representative images of SPP1 staining (a) and quantification (b) in bone metastasis lesions with treatment of vehicle or enzalutamide. (*P* values were computed by two-tailed unpaired Student's *t*-tests, ^∗^*P* < 0.05, ^∗∗^*P* < 0.01, and ^∗∗∗^*P* < 0.001).

To investigate the relationship between SPP1 expressions with enzalutamide resistance *in vivo*, we used the bone metastatic CRPC model developed by Prof. Qian lab, Queen's Medical Research Institute, University of Edinburgh, following intracardiac inoculation of an androgen-dependent murine prostate cancer cell line cells in FVB/N male mice. Through daily treatment with enzalutamide, bone metastatic CRPC model suffered from enzalutamide resistance. As the result of immunofluorescence staining (Figure 4), we found SPP1 was upregulated following enzalutamide resistance occurrence.

### 2.3. SPP1 Knockdown Enhanced the Sensitivity of CRPC Cell Lines to Enzalutamide

**Figure 5:**
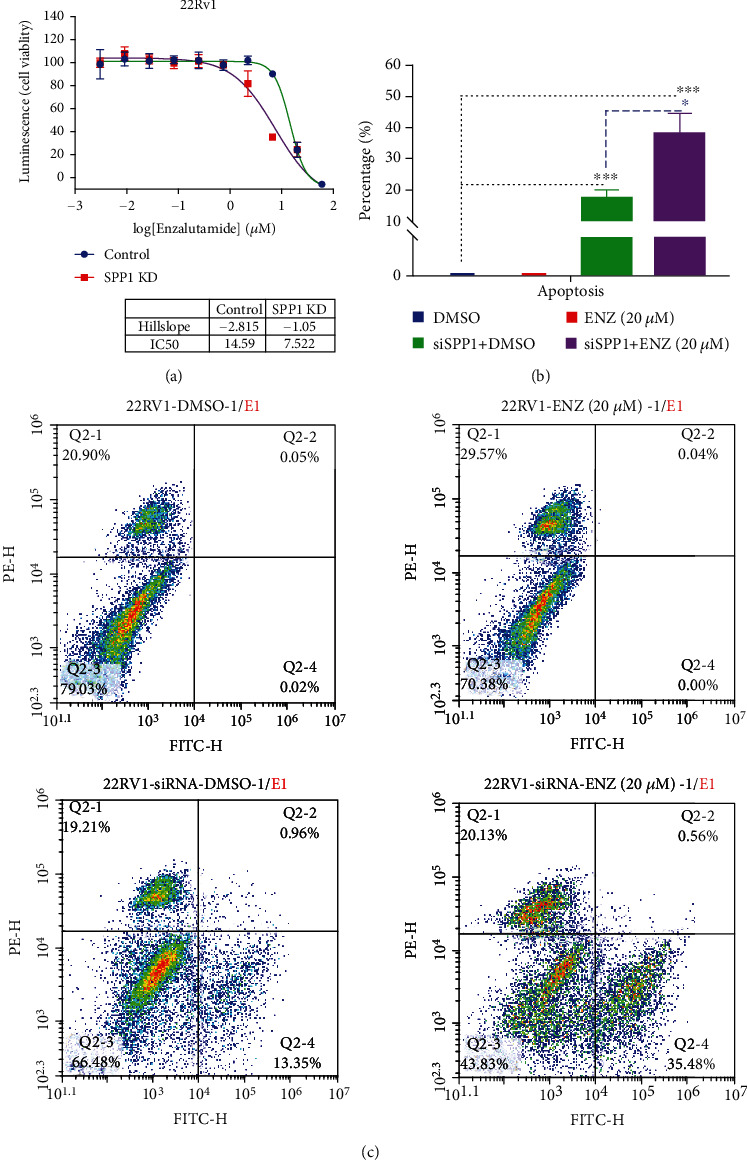
The effect of SPP1 knockdown on the sensitivity of CRPC cell lines to enzalutamide. (a) SPP1 knockdown significantly inhibited 22Rv1 cell proliferation after enzalutamide treatment and enhanced the enzalutamide sensitivity from IC50 = 14.59 to 7.522. (b, c) Cell apoptosis analysis demonstrated that SPP1 knockdown could significantly promote apoptosis rates and enhances apoptosis-promoting effect of enzalutamide. (*P* values were computed by two-tailed unpaired Student's *t*-tests, ^∗^*P* < 0.05, ^∗∗^*P* < 0.01, and ^∗∗∗^*P* < 0.001).

To clarify the role of SPP1 in the development of enzalutamide resistance in CRPC, 22Rv1 cell line with SPP1 knockdown was used to the effect of SPP1 on enzalutamide sensitivity. The results showed that SPP1 knockdown significantly inhibited 22Rv1 cell proliferation after enzalutamide treatment (Figure 5(a)). In addition, the rates of LNCaP cell proliferation following enzalutamide treatment were also assessed. Compared with that of SPP1-knockdown 22Rv1 cell treatment with enzalutamide, the IC_50_ value has no significant difference between SPP1-knockdown 22Rv1 and LNCaP, which was 7.522 and 8.650, respectively, which indicated SPP1 depleted can enhance enzalutamide sensitivity. In addition, result of cell apoptosis analysis (Figures 5(b) and 5(c)) also demonstrated that downregulated SPP1 by siRNA could significantly promote apoptosis rates (*P* < 0.001); more importantly, SPP1 knockdown remarkably enhanced apoptosis-promoting effect of enzalutamide (*P* < 0.001). Therefore, knockdown of SPP1 could induce the resensitization of enzalutamide-resistant CRPC cell line to enzalutamide.

### 2.4. The Role of PI3K/AKT Signaling in SPP1-Mediated Enzalutamide Resistant

**Figure 6:**
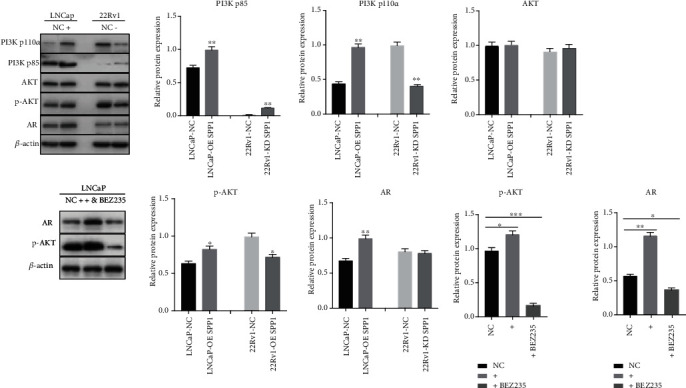
PI3K/AKT signaling activated by SPP1 in PCa cell lines. In SPP1 siRNA-transfected 22Rv1 cell lines, p-PI3K and p-AKT expression levels were decreased significantly. In contrast, p-PI3K and p-AKT were significantly upregulated in SPP1-overexpressed LNCaP cell line. And SPP1 overexpression was significantly upregulated AR expression in LNCaP cell line. Lentivirus-transfected LNCaP cells were treated with PI3K inhibitor BEZ235 (50 nM) for 48 h. PI3K inhibitor can reverse the effect of SPP1 overexpression on p-AKT and AR expression. (*P* values were computed by two-tailed unpaired Student's *t*-tests, ^∗^*P* < 0.05, ^∗∗^*P* < 0.01, and ^∗∗∗^*P* < 0.001.)

In our previous study, based on single-gene Gene Set Enrichment Analysis (GSEA) results of GEO database mining, we found SPP1 could regulate AR signaling pathway in the SPP1 high-expression CRPC group [13], which could be responsible for enzalutamide resistant. PI3K/Akt pathway plays an important role in the regulation of AR expression. Based on single-gene GSEA results, SPP1 could significantly regulated PI3K/AKT pathway in high-expression group (Supplement Figure 2). Western blot results (Figure 6) suggested that in the 22Rv1 cell line, SPP1 siRNA could repress p-PI3K and p-AKT expression levels significantly. In contrast, p-PI3K and p-AKT were significantly upregulated in SPP1 overexpressed LNCaP cell line. In addition, SPP1 was significantly upregulated AR expression in lentivirus-transfected LNCaP cell line. As the PI3K/AKT signaling pathway was activated abnormally in PCa, we evaluated the effect of PI3K inhibitor, BEZ235, on lentivirus-transfected LNCaP cell. PI3K inhibitor can reverse the effect of SPP1 overexpression on pAKT and AR expression, which suggested that SPP1 regulated AR expression by activating PI3K/AKT pathway. The results verified that the PI3K/AKT/AR signal pathway was remarkably activated by SPP1.

### 2.5. Knockdown of SPP1 Attenuated the Adhesion and Migration of CRPC Cells

**Figure 7:**
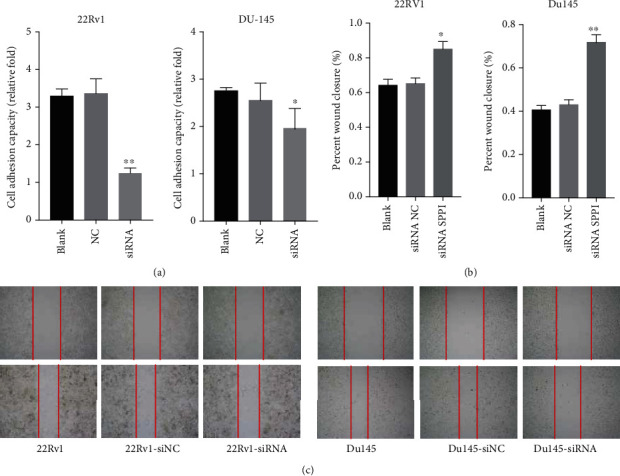
The effect of SPP1 knockdown on adhesion and migration of CRPC cells. (a) Matrigel transwell assay in SPP1 depleted and control groups. DU-145 and 22Rv1 adhesion was significantly decreased in SPP1 knockdown cell lines. (b, c) Wound closure assay in SPP1 depleted and control groups. An obvious decrease in the rate of wound closure was showed in the SPP1 siRNA-transfected group. (*P* values were computed by two-tailed unpaired Student's *t*-tests, ^∗^*P* < 0.05, ^∗∗^*P* < 0.01, and ^∗∗∗^*P* < 0.001).

Tumor cell adhesion to extracellular matrix proteins, for example, fibronectin, is important to provide a support for migration and metastasis. We next performed Matrigel transwell assay in SPP1 depleted and control groups (Figure 7(a)) for 48 h. DU-145 and 22Rv1 cells were used for this assay. Both DU-145 and 22Rv1 with SPP1 knockdown cell lines exhibited a significant decrease in adhesion on the fibronectin layer. The effects of knocking down SPP1 on migration of 22Rv1 and DU-145 cell lines were examined by wound closure assay. An obvious decrease in the rate of wound closure was showed in the SPP1 siRNA-transfected group (Figures 7(b) and 7(c)). Therefore, SPP1 knockdown significantly inhibited the migration and adhesion abilities of DU-145 and 22Rv1 cells.

### 2.6. SPP1 Activated EMT Pathway

**Figure 8:**
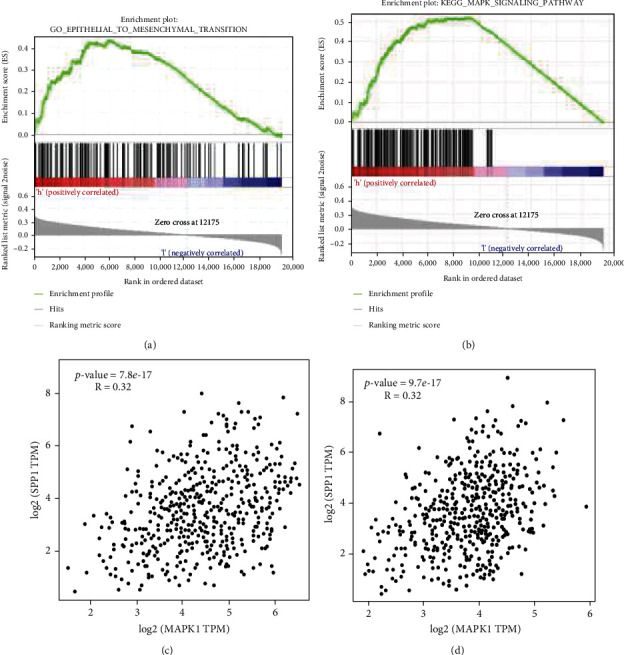
The single-gene GSEA of SPP1 and gene correlation analysis based on TCGA datamining. (a, b) SPP1 could significantly regulated EMT and MAPK pathway in the high-expression group. (c, d) Expression of MAPK1 and MAPK 14 was positively associated with that of SPP1 significantly.

**Figure 9:**
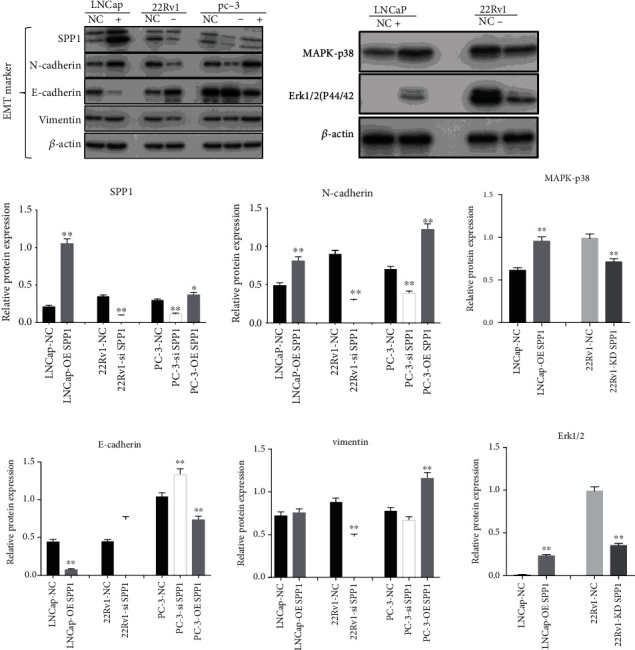
SPP1-mediated EMT pathway and ERK1/2 pathway activation. Upregulating SPP1 promoted, while silencing SPP1 attenuated EMT. SPP1 overexpression significantly improved the expression levels of MAPK-p38 and ERK1/2 in the lentivirus-transfected LNCaP cell, and SPP1 knockdown significantly attenuated MAPK-p38 and ERK1/2 expression. (*P* values were computed by two-tailed unpaired Student's *t*-tests, ^∗^*P* < 0.05, ^∗∗^*P* < 0.01, and ^∗∗∗^*P* < 0.001.)

EMT process plays a crucial role in tumor cell invasion and migration. In addition, after long-term treatment with enzalutamide, PCa cells undergo EMT changes, the epithelial markers in the tissue are significantly reduced, and the mesenchymal markers are significantly upregulated [16, 17]. As shown in Figure 6, the upregulation of AR expression is not obvious in the 22Rv1 siRNA-transfected group, which suggested SPP1 knockdown leading to the resensitization of enzalutamide was not due to downregulation of AR expression. EMT also acts an important driving factor for enzalutamide treatment resistance and disease recurrence. Based on single-gene GSEA results, SPP1 could significantly regulated EMT pathway in the high-expression group with a *P* value of 0.014 (Figure 8(a)). To clarify the regulation of SPP1 on EMT process, we assessed the expression of epithelial cell phenotype-specific proteins and mesenchymal cell phenotype-specific proteins in SPP1-knockdown 22Rv1, SPP1-overexpression LNCaP, and SPP1 overexpression and depleted PC-3 cell lines. As showed in Figure 9, protein expression of both vimentin and N-cadherin was obviously downregulated in siRNA-SPP1 22Rv1 (*P* < 0.01), compared with that in siRNA-NC ones. In contrast, vimentin and N-cadherin were significantly upregulated in lentivirus-transfected PC-3, compared with that in PC3-NC ones (*P* < 0.01). Specially, N-cadherin is remarkably downregulated in siRNA-SPP1 PC-3 (*P* < 0.01) and significantly upregulated in lentivirus-transfected LNCaP. Moreover, E-cadherin expression was both obviously upregulated in siRNA-SPP1 22Rv1 (*P* < 0.01) and PC-3 cell (*P* < 0.01), compared with that in siRNA-NC ones. Adversely, E-cadherin expression was both remarkably downregulated in lentivirus-transfected LNCaP (*P* < 0.01) and PC-3 (*P* < 0.01). These results indicated that SPP1 could activate EMT pathway in PCa cells.

### 2.7. MAPK/ERK1/2 Pathway Activation Associated with SPP1-Mediated EMT

The MAPK/ERK pathway plays major roles in regulating tumor cell proliferation and migration. To reveal the molecular mechanisms in EMT progression, we assessed SPP1-mediated MAPK/ERK pathway activation. As showed in Figure 8(b), the single-gene GSEA analysis of TCGA showed that SPP1 could significantly regulated MAPK pathway with a *P* value of 0.004, and expression of MAPK1 and MAPK14 was positively associated with that of SPP1 significantly (Figures 8(c) and 8(d)). MAPK1 and MAPK14 encode extracellular signal-regulated kinase 2 (ERK1/2) and MAPK p38, which play important roles in the cascades of cellular responses induced by extracellular stimuli leading to transcription factors activation directly. We found that SPP1 overexpression significantly increased the expression levels of MAPK-p38 and ERK1/2 in the lentivirus-transfected LNCaP cell, and in contrast, SPP1 knockdown significantly decreased MAPK-p38 and ERK1/2 expression (Figure 9).

## 3. Discussion

PCa is cumulatively becoming a major health threat in men worldwide. Due to lack of reliable biomarkers for predicting the malignant progression of PCa cells, there is a bottleneck in its accurate diagnosis, prognosis, and estimate of the existing therapy effectiveness. In addition, bone metastasis and antiandrogen drug resistance are the key problems for PCa treatment and the main cause of PCa mortality. Therefore, it is of great importance to assess the prognostic potential of novel signatures involved in the malignant progression of PCa and identify the metastasis and drug resistance related biomarkers or targets. SPP1, known as a multifunctional protein, is a well-characterized ligand for the alphavbeta3 integrin [7]. Integrin receptors belong to heterodimeric transmembrane glycoproteins, which binds to SPP1 in an RGD-dependent way regulating tumor cell adhesion, migration, proliferation, and survival [19]. Our computational results and previous studies suggested that SPP1 was highly expressed in numerous cancers and its expression level correlates with the metastatic level of several tumors. But the clinical significance and biological roles of SPP1 in tumor metastasis are still incompletely understood.

In a previous study, we identified SPP1 as one of the most abundantly EMC genes in mCRPC, which was further confirmed that SPP1 was overexpressed both at the computational and IHC levels. Computational comparisons of different prostate samples suggested an increased expression of SPP1 in association with the progression and prognosis of CRPC, indicating its potential value as a biomarker for CRPC progression. IHC analysis was applied to discover the origin of SPP1 expression in primary PCa and CRPC with bone metastasis samples. Our findings were in agreement with previous data which suggested that SPP1 were mainly released from tumor cells and was highest expressed in bone metastatic site. Although SPP1 was expressed in all four PCa cell lines, SPP1 expression level was lower in the androgen-dependent PCa cell line, LNCaP, than in 22Rv1 cell line, which is enzalutamide-resistant PCa cell. The progressively malignant CRPC cell lines of DU-145 and PC-3 have a high expression of SPP1 in both RNA and protein levels. In addition, in the bone metastatic CRPC mice model, we found SPP1 were significantly associated with enzalutamide resistance. Together, these data indicated that SPP1 indeed highly expressed in CRPC cell lines and tissues and suggested its potential role in antiandrogen resistance and metastatic CRPC progression.

Although enzalutamide improves the overall efficacy in treating CRPC, the eventual development of resistance is mostly inevitable [20]. In the cell proliferation and apoptosis, SPP1 knockdown has been observed to inhibit proliferation of 22Rv1 and enhance apoptosis-promoting effect of enzalutamide, which suggested SPP1 knockdown could induce the resensitization of enzalutamide-resistant CRPC cell line to enzalutamide. The PI3K/AKT/AR signaling pathway plays an important role in the variability in response to enzalutamide and antiandrogen drug resistance [21]. Numerous experiments indicated that a reciprocal relationship exists between the AR and PI3K/AKT pathway [22, 23]. In addition, PI3K or AKT inhibitors were studied in clinical trials [23]. In our study, p-PI3K and p-AKT expression levels declined after SPP1 knockdown, so SPP1 siRNA could inhibit the PI3K/Akt signal pathway in PCa cells. In addition, SPP1 overexpression could promote AR expression in LNCaP cell line, which might promote antiandrogen drug resistance.

In our study, we also found knockdown of SPP1 could attenuate the adhesion and migration of CRPC cells, downregulate vimentin and N-cadherin, and upregulate E-cadherin in CRPC cell lines, which suggested that SPP1 promoted metastasis by activating EMT pathway in CRPC. SPP1 overexpression could promote EMT activation. EMT as an imperative phenotypic conversion primarily occurs at the onset of invasion by reducing intercellular adhesion and enhancing motility. In addition, abnormal activation of EMT was involved in CRPC bone metastasis [24, 25]. In this study, we confirmed that SPP1 promoted CRPC metastasis by activation of EMT. Several signaling pathways, such as Erk1/2, TGF-beta, and Akt, trigger EMT responses [15]. Emerging evidence indicates that ERK is involved in TGF-*β*-mediated EMT. In addition, ERK pathway activation induced by RAS or RAF also engages in EMT, and ERK1/2 blockade inhibits EMT in lung cancer cells, which suggests that ERK1/2 functions as an EMT inducer [26]. In this study, we found SPP1 could trigger the ERK1/2 signaling pathway, which might demonstrate potential molecular mechanism of SPP1-mediated EMT progression.

## 4. Materials and Methods

### 4.1. Bioinformatics Analysis of the Clinical Significance of SPP1

FPKM gene expression data, somatic mutation, and survival data of Pan-cancer analyzed in The Cancer Genome Atlas (TCGA) project were downloaded from University of California Santa Cruz (UCSC) Xena website (https://xenabrowser.net/datapages/) [27]. Tumor mutational burden (TMB) was computed according to total number of mutations referring to all nonsynonymous mutations in the coding region and mutations at splice sites [28]. The R package survivalROC was utilized to study the time-dependent prognostic value of SPP1. A two-sided log-rank *P* < 0.05 was considered significance for survival analysis. The relationship of SPP1 expression level with individual cancer stages and lymph node metastasis status was analyzed by UALCAN database (http://ualcan.path.uab.edu/) [29], which is portal for the relationship among gene expression, tumor subgroup, and survival analyses. SPP1 expression in different metastatic sites was analyzed in Human Cancer Metastasis Database (HCMDB, http://hcmdb.i-sanger.com/) [30], which is an integrated database of Gene Expression Omnibus (GEO) and TCGA designed to reserve and process large scale expression data in tumor metastasis. GSE32269 from GEO database totally including hormone sensitive prostate cancer and CRPC samples were processed using the DESeq package in R.

### 4.2. Cell Culture and Transfections

PC-3, DU-145, LNCaP, and 22Rv1 were obtained from Cell Bank of Peking Union Medical College Culture Collection (Beijing, China). LNCaP and 22Rv1 cell lines were cultured in RPMI-1640 medium with 10% fetal bovine serum, and 100 mg/mL penicillin and 100 mg/mL streptomycin were used to culture the cells. PC-3 and DU-145 cell line was cultured in F-12K Medium and Eagle's Minimum Essential Medium, respectively. Cell culture was at 37°C with 5% CO_2_-humidified atmosphere. RNA interference was applied to knockdown SPP1 in cell lines. In our study, three different SPP1siRNAs and lentiviral vector were synthesized by JTS scientific (Wuhan, China). All these siRNAs were transfected into PCa cell lines by Lipofectamine 8000 based on the product manual (Invitrogen). The following siRNA sequences were finally used: SPP1(H)-508 (sense oligo: 5′-GAGUUGAAUGGUGCAUA-CATT-3′, antisense oligo: 3′-UGUAUGCAC CAUUCAACUCTT-5′). SPP1 (target sequence, NP_001035149.1) was cloned into the lentiviral vector JLVO-CAG-GFP-Apuro. Lentiviral vector was transfected into PC-3 and LNCaP cell lines with polybrene, and cell lines with stable SPP1 expression were selected and amplified.

### 4.3. RNA Isolation and qRT-PCR

Easy pure RNA Kits (TransGen Biotech, BJ, China) were applied for total RNA extraction. TransScript First-Strand cDNA Synthesis SuperMix (TransGen Biotech, BJ, China) was used for reverse transcription according to the manufacturer's protocol. The mRNA expression level was acquired through cyclic threshold method. SPP1 primers were used: SPP1 (5′-GTTAAACAGGCTGATTCTGG-3′ (forward), 5′-CATGGTCATCATCATCTTCA-3′ (reverse)). PCR reactions were performed in triplicates with following context: after an initial hot start at 95°C for 5 min, PCR amplification was conducted for 40 cycles of 95°C/10 s and 60°C/30 s, denaturation at 95°C/15 s, and annealing and extension at 60°C for 60 s. The relative level of gene expression was detected via the cyclic threshold method.

### 4.4. Western Blot

The protein expression levels of SPP1, vimentin, E-cadherin, N-cadherin, MAPK p38, ERK1/2, PI3K p110*α*, PI3K p85, AKT, pAKT, AR, and *β*-actin were evaluated by Western blot. 2.5 × 10^5^ cells were plated in 6-well plates at 37°C with 5% CO2 for 24 h and were washed thrice in ice-cold PBS and harvested, and cell pellets were resuspended in NP-40 lysis buffer (Beyotime, P0013F) containing protease inhibitors and subjected to sonication. Protein concentrations were assessed using a Pierce bicinchoninic acid protein assay kit (Beyotime, P0006.). Equal amounts of proteins (20 *μ*g per sample) were separated by SDS-PAGE and transferred onto PVDF membranes. After being blocked with 5% milk in TBST, the membrane was incubated at 4°C overnight with the primary antibody (1 : 1,000). The following primary antibodies were used: anti-SPP1 (Abcam, Cambridge, UK), anti-vimentin (Cell Signal Tech, Danvers, USA), anti E-cadherin (Cell Signal Tech, Danvers, USA), anti-N-cadherin (Cell Signal Tech, Danvers, USA), anti-MAPK p38 (Cell Signal Tech, Danvers, USA), anti-ERK1/2 (Cell Signal Tech, Danvers, USA), anti-PI3K p110*α* (Cell Signal Tech, Danvers, USA), anti-PI3K p85 (Cell Signal Tech, Danvers, USA), anti-AKT (Cell Signal Tech, Danvers, USA), phopho-AKT (Ser 473) (Cell Signal Tech, Danvers, USA), anti-AR (Cell Signal Tech, Danvers, USA), and anti-*β*-actin (Proteintech, Chicago, USA). Subsequently, the membranes were washed thrice with PBS and incubated with horseradish peroxidase-conjugated secondary antibodies (anti-rabbit or anti-mouse IgG; 1 : 5,000; Cell Signaling Technology, Inc.). Bands were visualized with BeyoECL Plus (Beyotime, P0018S). BEZ235 was obtained from Med Chem Express (Monmouth Junction, USA).

### 4.5. Immunohistochemical Staining

Immunohistochemical (IHC) staining was performed to determine the expression of SPP1 in primary PCa and bone metastasis CRPC. The details of IHC staining procedure are similar to those described in our previous study [13]. The slices were immersed in methanol with 3% hydrogen peroxide, after dewaxing in xylene and rehydrating in ethanol, then washed in tap water, and then immersed in distilled water. The slides were incubated with anti-SPP1 antibody (Abcam, Cambridge, UK) in PBS at a dilution of 1 : 400 at 4°C overnight and then incubated with the secondary antibody. Immunofluorescence staining with paraffin embedded parts was performed similarly with immunohistochemistry staining using anti-SPP1 antibody (1 : 400). Next, sections were incubated AF555-conjugated secondary Abs and counterstained with DAPI for nucleus. Staining of SPP1 was quantified as the area of positive signals divided by the filed area using ImageJ.

### 4.6. Cell Proliferation Assay

Through the manufacturer's protocol, the cell proliferation test was performed by using the Celltiter-Glo assay (Promega P/N G7570). In this assay, the luminescence value is directly proportional to the amount of adenosine triphosphate (ATP), and ATP is positively related to the number of living cells, so cell viability can be obtained by detecting the ATP content. The cell lines were transfected with siRNA SPP1 or siRNA negative control (NC) and disposed by enzalutamide. Every 24 h, viable cell counts were detected, and both the SPP1 siRNA and siRNA-NC groups were conducted independently in triplicate.

### 4.7. Cell Apoptosis Assay

22Rv1 and its siRNA SPP1-transfected cell lines were plated in 6-well plates at 3 × 10^5^/well. Four groups were involved in this experiment: the control group disposed with DMSO, enzalutamide treatment group, siRNA SPP1-transfected group, and SPP1 knockdown with enzalutamide treatment group. All the groups were stained with Annexin V and PI (BD Biosciences, CA, US). Afterwards, the stained cells were examined by a flow cytometer (BD Biosciences) and data were processed by Cell Quest software. All the experiments were carried out in triplicate.

### 4.8. Migration and Adhesion Assay

Migration ability of cells with or without SPP1 transfected was evaluated by wound healing assays. Monolayers of PCa cell lines and their transfected cells were cultured in 24-well plates. Cell layer was scratched with a 200 *μ*L pipette's tip and washed several times using medium to wash dislodged cells. DU-145, 22Rv1, and their transfected cell lines migrated into wound area were photographed at the 48 h and 72 h, respectively.

After being transfected with SPP1 siRNA or siRNA NC, the cell lines were cultured with 1% fetal bovine serum and plated into the top chamber; cell invasion abilities were evaluated by Matrigel transwell assays. 5 × 10^4^ cells were added into the top chamber of a polycarbonate transwell filter chamber covered with Matrigel. After 48 h, the cells in the upper of membrane were wiped off and fixed with 4% paraformaldehyde and stained with 0.5% crystal violet for counting the number of cell. All experiments were carried out in triplicate.

### 4.9. Statistical Analysis

GraphPad Prism 8.0 (GraphPad Software, Inc., CA, USA) was applied for statistical analysis. Data are showed as the mean ± S.E.M. Two groups were compared using Student's *t*-test. *P* value < 0.05 was defined as significant.

## 5. Conclusions

Our current data illustrated for the first time that overexpression of SPP1 in PCa bone lesions and enzalutamide resistance CRPC *in vitro* and *in vivo*. SPP1 could promote PCa cell metastasis by activating EMT, and we firstly reported that SPP1 could be a possible target against enzalutamide resistance and EMT in CRPC treatment, which could be regulated via the MAPK/ERK1/2 and PI3K/AKT pathways. This indicated that specific targeting of SPP1 can be a valid approach to treat antiandrogen resistance of metastatic CRPC.

## Data Availability

`The available public datasets from GEO database (https://www.ncbi.nlm.nih.gov/gds) and TCGA project from University of California Santa Cruz (UCSC) Xena website (https://xenabrowser.net/datapages/) were downloaded and analyzed in this study. Gene Set Enrichment Analysis (GSEA) was performed using GSEA 2.2.1 (http://www.broadinstitute.org/gsea). UALCAN database (http://ualcan.path.uab.edu/) and Human Cancer Metastasis Database (HCMDB, http://hcmdb.i-sanger.com/) were applied to investigate the clinical significance of SPP1.

## Supplementary Materials

Supplementary Materials Supplement Figure 1: metastasis-free survival analysis of SPP1 based on TCGA database. Supplement Figure 2: the single-gene GSEA of SPP1 on PI3K/AKT pathway based on TCGA datamining.
