# Supplementary material for: SPP1 Promotes Enzalutamide Resistance and Epithelial-Mesenchymal-Transition Activation in Castration-Resistant Prostate Cancer via PI3K/AKT and ERK1/2 Pathways
Source: Oxid Med Cell Longev. 2021 Oct 22;2021:5806602. doi: 10.1155/2021/5806602 (PMC8556132; doi:10.1155/2021/5806602)
Supplement: Supplementary Materials — Supplement Figure 1: metastasis-free survival analysis of SPP1 based on TCGA database. Supplement Figure 2: the single-gene GSEA of SPP1 on PI3K/AKT pathway based on TCGA datamining. [file 5806602.f1.docx]

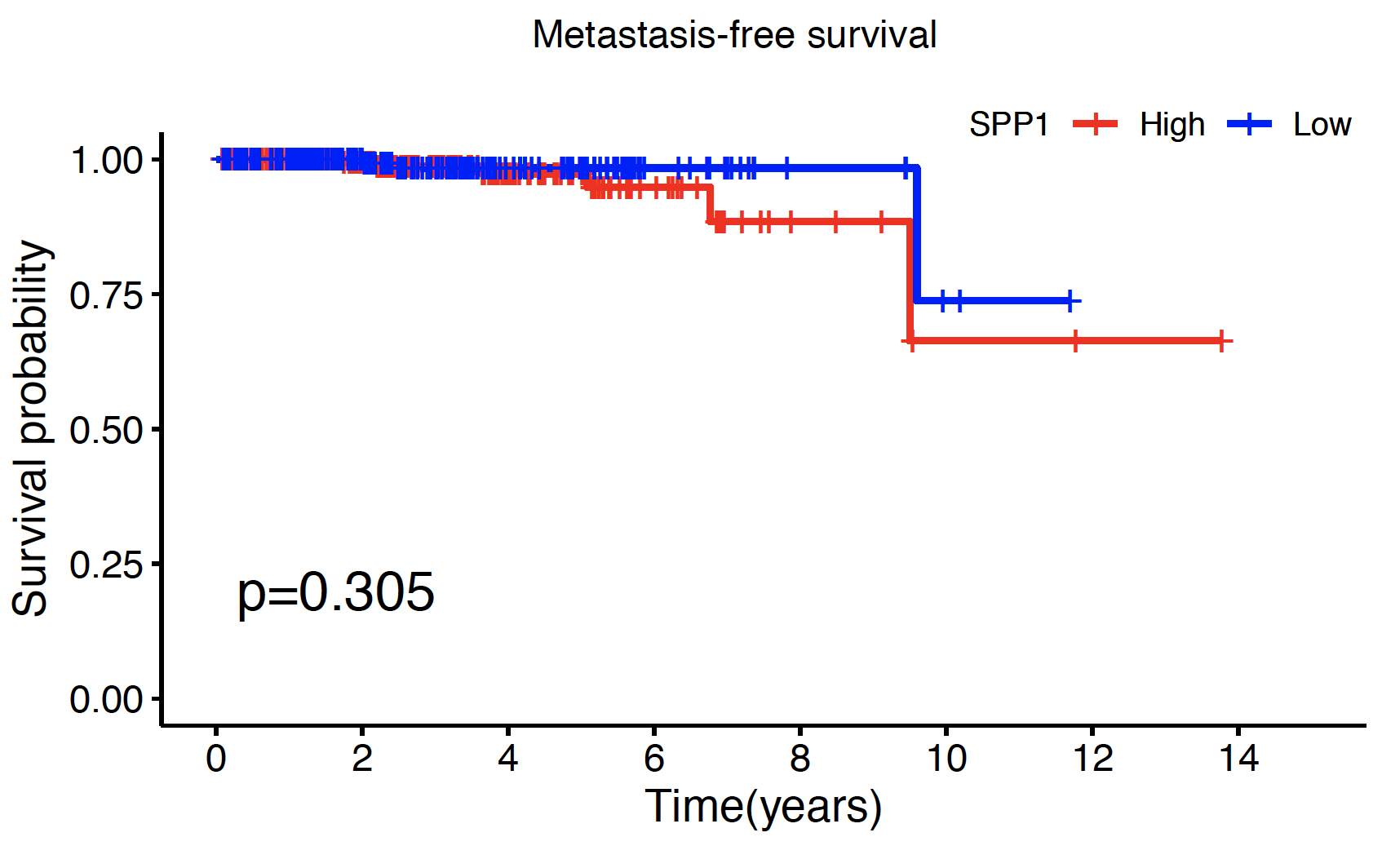


Supplement Figure 1. Metastasis-free survival analysis of SPP1 based on TCGA database.


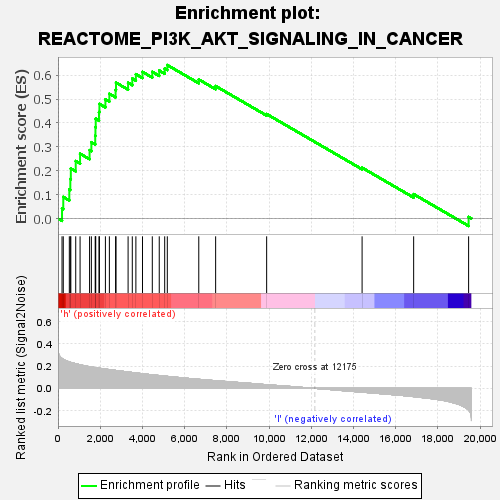


Supplement Figure 2. The single-gene GSEA of SPP1 on PI3K/AKT pathway based on TCGA datamining.
